# Supplementary material for: Phytosterol-Loaded Surface-Tailored Bioactive-Polymer Nanoparticles for Cancer Treatment: Optimization, In Vitro Cell Viability, Antioxidant Activity, and Stability Studies
Source: Gels. 2022 Apr 2;8(4):219. doi: 10.3390/gels8040219 (PMC9026838; doi:10.3390/gels8040219)
Supplement: Supplementary file 1 [file gels-08-00219-s001.zip › gels-1599941-supplementary.pdf]

gels

MDPI

Supplementary Materials

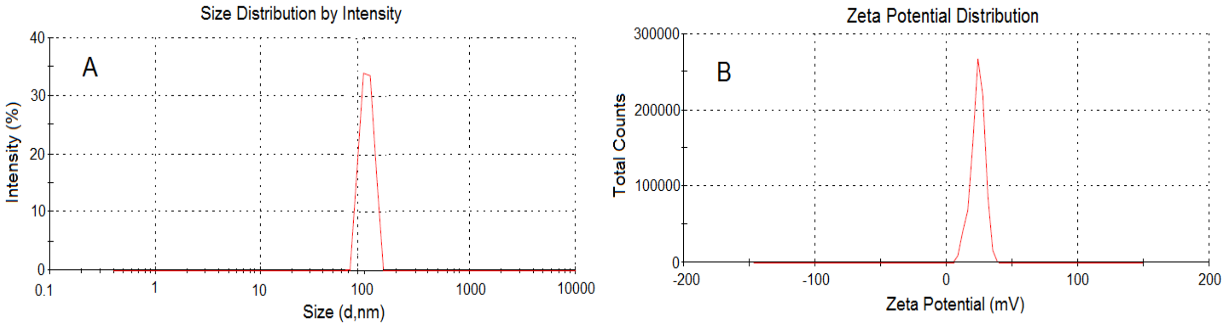

Figure S1. Particle size distribution curve from (**A**), and Surface charge as zeta potential (mV) of β-SIT-Alg/Ch-NPs-FA (**B**).

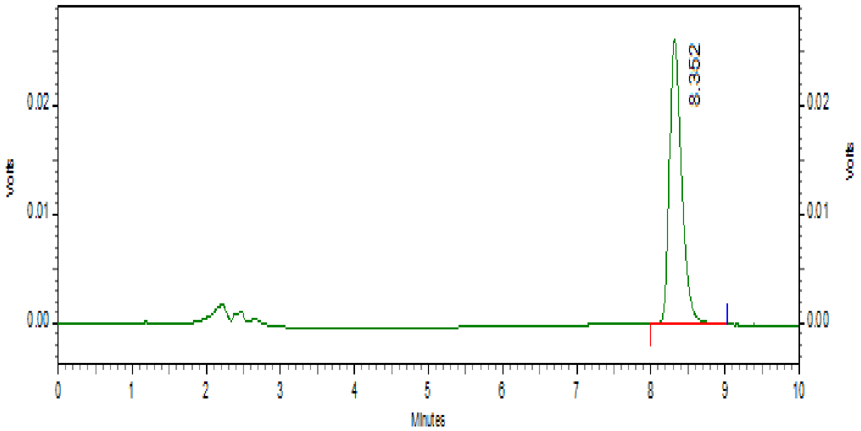

Figure S2. A representative chromatogram of β-SIT.
